# Cerebral organoids as a platform to model prenatal exposure to drugs of abuse

**DOI:** 10.4103/NRR.NRR-D-25-01200

**Published:** 2026-01-27

**Authors:** Louise Keegan, Kate Montwill, Ciaran Kennedy, Ciara L. McMahon, Jessica L. Davis, Keith J. Murphy

**Affiliations:** Neurotherapeutics Research Group, School of Biomolecular and Biomedical Science, Conway Institute, University College Dublin, Belfield, Dublin, Ireland; School of Biomolecular and Biomedical Science, Conway Institute, University College Dublin, Belfield, Dublin, Ireland

Substance abuse is a global public health concern that, over the past decade, has increased by 23% worldwide (UNODC, 2023). The number of people who suffer from drug use disorders in 2023 was estimated to be 39.5 million, a 45% increase over the 10 years since 2013 (UNODC, 2023). Related to this increase, approximately 5% of pregnant women now report using one or more addictive substances during pregnancy, highlighting prenatal exposure to drugs like opioids, alcohol, and cocaine as a significant global health concern with serious consequences for fetal neurodevelopment and long-term mental health (NIDA, 2020). *In utero* exposure to drugs of abuse can alter brain development trajectories, leading to lifelong cognitive, behavioral, and psychiatric issues. Such *in utero* exposure is thought to disrupt early processes such as neurogenesis, gliogenesis, and cortical organization, ultimately resulting in structural and functional deficits. Until recently, progress in this research area has relied on either very limited human studies, typically involving correlative observation, or animal models. These studies are constrained by ethical considerations and interspecies differences, particularly relating to brain complexity and developmental timing.

Recent advances in stem cell biology have facilitated the creation of three-dimensional (3D) human cerebral organoids (COs), which mimic key features of early brain development. Unlike traditional *in vitro* systems, organoids provide a cytoarchitecturally relevant environment where neuronal differentiation, migration, and synaptogenesis can be observed in real time. Thus, COs present a unique, human-specific, and ethical platform to model the effects of prenatal drug exposure.

Here, we explore the use of COs to model prenatal exposure to drugs of abuse such as opioids, alcohol, and cocaine. We highlight the convergent molecular and cellular disruptions of neurodevelopment that have been revealed to be shared across many drugs of abuse. We argue that CO models provide the first experimentally tractable, human-specific system to connect these molecular and cellular perturbations with early network dysfunction. They can be harnessed to test reversibility toward the development of novel therapeutic strategies.

**Cerebral organoids as tools for neurodevelopment research:** Cerebral organoids represent a significant advancement in developmental neurobiology research, offering a physiologically relevant and human-specific model system (Notaras et al., 2021; Li et al., 2025). Generated from embryonic or induced pluripotent stem cells, these 3D cultures self-organize to form discrete brain regions, recapitulating aspects of early human brain architecture, including ventricular-like zones, radial glia, and developing cortical layers. Crucially, organoids exhibit region-specific gene expression patterns, allowing for the exploration of developmental trajectories that two-dimensional cultures or rodent models do not adequately address.

Cerebral organoids capture progenitor dynamics, including radial glia and outer radial glial cell (oRGC) proliferation, fate choice, and apoptosis. Axon/neurite outgrowth, synaptogenesis, and nascent network activity can all be measured, allowing an assessment of functional maturation. Importantly, perturbations can be mapped from gene regulatory changes to connectivity in the same specimen. Moreover, COs enable the study of human-specific features, such as prolonged neurogenesis, the expansion of oRGCs, cortical folding potential, and prolonged synaptic maturation.

Organoids also facilitate experimental designs that would be unethical or impossible in human subjects, such as the chronic exposure of early-stage neuroepithelial tissues to teratogens. Chronic or pulsed drug exposures can be tightly aligned to discrete developmental windows (such as neuroepithelial, ventricular-zone expansion, and onset of synaptogenesis), with dose–response mapping and washout designs to test reversibility of any deficits induced. These capabilities make organoids particularly suitable for studying how exposures to drugs of abuse perturb human neurodevelopment at the cellular and molecular levels.

Importantly, organoids can be derived from induced pluripotent stem cells obtained from genetically diverse donors, enabling the study of interindividual variation in susceptibility to drug-induced neurotoxicity. Such genetic influences can be further dissected using powerful techniques like CRISPR-based gene editing. Further complexity can be added through assembloids, multi-region organoid systems that model inter-regional connectivity, or co-culture systems incorporating microglia, vascular elements, and maternal-fetal interfaces. These innovations extend the relevance of organoid platforms, setting the stage for comprehensive modeling of prenatal drug exposures (**Table 1**).

**Table 1:** Advantages of cerebral organoids for prenatal substance exposure research

Neurodevelopmental/prenatal exposure-related processes	Representative cerebral organoid readouts/assays	Example findings from opioid, alcohol, and cocaine exposure models (see text for references)	Mechanistic insights enabled
Stem cell niche dynamics	Cell-state mapping by scRNA-seq; EdU/Ki67 proliferation indices; cleaved-caspase-3; radial glia/outer radial glial cell quantification	Fentanyl exposure stalls progenitor cell pool; ethanol reduces outer radial glial cell numbers and increases apoptosis; cocaine alters progenitor proliferation	Defines early cellular bottlenecks leading to cortical thinning and neuron-glia imbalance
Tissue organization & polarity	Immunostaining for apical–basal markers (ZO-1, PAX6, and TBR2); 3D morphometrics of ventricular-like zones	Ethanol disrupts polarity and neuroepithelial organization; opioid exposure alters extracellular matrix program	Links extracellular and polarity defects to defective cortical layering
Synaptogenesis & network maturation	Synapse gene-module analysis; synaptic puncta imaging; calcium imaging or multielectrode array recordings	Opioid exposure down-regulates synaptogenesis; ethanol reduces spontaneous network activity	Connects molecular synaptic failure with functional hypoactivity
Inflammation & oxidative stress	Bulk/single-cell transcriptomics; glial activation markers; reactive oxygen species assays	Cocaine exposure triggers reactive oxygen species accumulation and astrocyte activation; opioids induce inflammatory gene expression	Reveals convergent stress-response programs driving progenitor loss
Transcription & epigenomic remodeling	Single-cell RNA-sequencing, Assay for Transposase-Accessible Chromatin using sequencing, Cleavage Under Targets and Tagmentation, profiling over time	Cocaine produces persistent chromatin accessibility and transcriptional changes post-withdrawal	Demonstrates durable molecular “memory” of prenatal exposure
Circuit integration (assembloids)	Region-specific cerebral organoid combination (cortex–striatal–midbrain); axon-projection tracing; long-range connectivity assays	Cocaine perturbs dopaminergic projection formation and transcriptional signatures	Enables circuit-level analysis of addiction-related connectivity and recovery potential
Therapeutic response & screening	Drug-treatment rescue paradigms; high-content imaging; multi-omics and activity endpoints	Insulin-like growth factor 1 rescues ethanol-induced network deficits; candidate targeting of transforming growth factor beta 1 in prenatal opioid exposure	Supports mechanism-based screening of therapeutic compounds
Inter-individual variability & personalization	Induced pluripotent stem cells donor diversity; CRISPR perturbation of risk loci; comparative omics	Possible variable susceptibility to progenitor injury across donor lines	Precision-risk stratification and personalized intervention testing

**Cerebral organoids in the study of prenatal opioid exposure:** The opioid epidemic has resulted in increased urgency to understand how prenatal opioid exposure affects fetal brain development. Clinical studies associate prenatal opioid exposure with increased risk of neonatal abstinence syndrome, reduced cognitive function, attention-deficit/hyperactivity disorder, and heightened susceptibility to psychiatric disorders later in life. The precise mechanisms, however, by which opioids alter neurodevelopment have been challenging to elucidate due to species differences in neurogenesis timing, opioid receptor expression, and placental metabolism.

Studies using morphine, oxycodone, buprenorphine, fentanyl, and methadone in organoids demonstrate that chronic opioid exposure disrupts multiple neurodevelopmental processes. (Ho et al., 2022; Dwivedi et al., 2023; Yao et al., 2023; Kim et al., 2024). These studies have revealed impaired growth of COs exposed to methadone, suggestive of impaired cortical neurogenesis and/or increased apoptosis. Similarly, cell type proportional analysis in midbrain organoids showed that the potent opioid fentanyl stalled neurogenesis at a progenitor cell stage, leading to a reduction in the production of mature neurons (Kim et al., 2024).

CO studies have also shown that opioid exposure leads to reductions in neuronal activity, which is strongly suggestive of impairments in the maturation of synaptic connectivity. Bulk and single-cell RNA sequencing studies reveal opioid-mediated transcriptional dysregulation of genes involved in interferon signaling, synaptogenesis, extracellular matrix, and ciliary function (Ho et al., 2022; Dwivedi et al., 2023). This transcriptional disruption is driven by dysregulation of a growth factor and matricellular signaling regulatory network centered around transforming growth factor beta 1 and thrombospondin 1, strongly suggestive of a disruption in synapse formation (Dwivedi et al., 2023). These effects appear to be principally mediated through µ-opioid receptor signaling. Interestingly, µ-opioid receptors are expressed to a significantly higher degree in the human progenitor zone compared to rodents, again underlining the importance of human-specific model systems.

**Cerebral organoid studies of fetal alcohol spectrum disorders:** Alcohol is a potent teratogen and the leading preventable cause of neurodevelopmental impairment globally. Fetal alcohol spectrum disorders affect an estimated 1%–5% of children worldwide (UNODC, 2023), and are characterized by outcomes including microcephaly, reduced cortical thickness, behavioral dysregulation, and impaired executive function. Disruptions in radial glial cells (RGCs), which serve as both progenitors and scaffolds for neuronal migration during cortical development, may underpin such neurodevelopmental deficits.

Human COs have been instructive in researching the cellular basis of ethanol-induced neurotoxicity (Arzua et al., 2020; Adams et al., 2023; Lu et al., 2023). When exposed to ethanol, forebrain organoids exhibit a clear structural disruption of radial glial morphology and polarity, as evidenced by reduced expression of markers such as nestin, GFAP, and vimentin (Lu et al., 2023), which impairs the orderly formation of cortical layers. Moreover, ethanol exposure has been shown to reduce the population of oRGCs, which are abundant in human brains but not in rodent brains, and are critical for neocortical expansion. Specifically, ethanol-exposed organoids exhibit fewer HOPX^+^ oRGCs and diminished proliferation, which might contribute to cortical thinning. Increased apoptosis within the progenitor zones has also been observed and would be predicted to compound developmental defects further. In parallel, there is an increase in the number of cells expressing the astrocytic markers, GFAP and S100β, suggesting a premature switch from neurogenesis to gliogenesis. This shift would reduce the pool of neurons generated and alter the cellular composition of the developing cortex.

At the molecular level, ethanol has been shown to alter chromatin accessibility and histone post-translational modification patterns related to transcriptional regulation of genes involved in neurogenesis and synaptic development (Adams et al., 2023). These molecular changes were accompanied by reduced neuronal network activity, which, interestingly, was partially rescued by insulin-like growth factor 1, suggesting that alcohol-induced neuronal hypoactivity, like that induced by opioids, relates to a deficit in trophic support.

These mechanistic insights provide a plausible explanation for clinical observations in fetal alcohol spectrum disorders, including reduced cortical volume and executive dysfunction. Moreover, organoid models provide a platform for investigating potential interventions which may mitigate ethanol-induced damage.

**Prenatal cocaine exposure cerebral organoid research:** Prenatal cocaine exposure has long been associated with a spectrum of adverse neurodevelopmental outcomes, including deficits in executive function, attention, and emotional regulation. These effects vary with the timing, dose, and duration of exposure and can be compounded by social and environmental factors postnatally. Cocaine’s lipophilic nature enables it to readily cross the placenta and blood–brain barrier, exposing the fetal brain to high concentrations of the drug during critical stages of development.

Unlike opioids or ethanol, cocaine primarily exerts its neurotoxic effects through the inhibition of monoamine reuptake transporters, leading to elevated extracellular dopamine, norepinephrine, and serotonin. In the developing brain, however, where these systems are still emerging and maturing, the effects of cocaine are likely also mediated through additional mechanisms, including sodium channel blockade and induction of oxidative stress.

Studies using cortical and ventral forebrain organoids have shown that cocaine impairs progenitor proliferation and accelerates premature neuronal differentiation. CYP3A5-mediated production of reactive oxygen species seems to be a central mechanism involved. Importantly, reactive oxygen species scavenging was shown to rescue much of the observed cocaine-mediated pathology (Lee et al., 2016). Moreover, cocaine exposure activates reactive oxygen species-dependent inflammatory pathways, reinforcing oxidative stress as a conserved mechanism of injury (Rudibaugh et al., 2023).

More recent single-cell RNA-sequencing and epigenomic analyses of cocaine-exposed organoids have confirmed widespread transcriptional dysregulation. Specifically, upregulation of genes involved in inflammation was observed, particularly in astrocyte progenitor cell populations (Davis et al., 2025). Moreover, cocaine altered the epigenetic landscape across various cell types, underpinning dysregulated expression of immediate early genes and synapse-associated genes. These alterations are linked to aberrant trophic signals, such as increased levels of midkine produced by astrocyte precursor cells that have been activated by cocaine exposure (Davis et al., 2025).

In assembloid models incorporating midbrain, striatum, and cortex, chronic cocaine exposure perturbs long-range dopaminergic projections and persistently alters gene expression, even following drug withdrawal (Reumann et al., 2023). This suggests that the effects of cocaine may extend beyond development into long-term circuit maturation and function. Collectively, these findings demonstrate that cerebral organoids facilitate the elucidation of both cellular and network-level consequences of prenatal cocaine exposure, thereby providing a bridge between molecular pathology and clinical neurodevelopmental outcomes.

**Figure 1:**
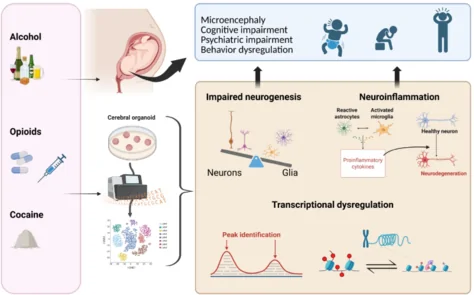
Graphical summary of the perspective. Prenatal exposure to drugs of abuse like alcohol, opioids or cocaine can disrupt neurodevelopmental processes leading to later emergence of neuropsychiatric disorders and cognitive impairments. Human cerebral organoids recapitulate early brain development, including progenitor dynamics, neuronal differentiation, synaptogenesis, and emerging network activity and can be used to model prenatal exposure to such drugs of abuse. Such studies have already revealed impaired neurogenesis, apoptosis, oxidative stress, neuroinflammation, and reduced neuronal activity as key mechanisms behind the detrimental effects of drugs of abuse on neurodevelopment. These powerful organoid models of human brain development can reveal deep insight into the transcriptional and epigenetic perturbations that underlie drug-induced dysregulation of neurodevelopment. Created with BioRender.com.

**Shared and substance-specific mechanisms:** Despite their pharmacological differences, opioids, alcohol, and cocaine elicit convergent effects on early brain development when modeled using cerebral organoids (**Figure 1**). A recurring theme across substances is the disruption of neurogenesis. Each drug interferes with neural progenitor proliferation, induces premature differentiation and increases apoptosis, leading to a depleted or stalled progenitor pool and reduced numbers of maturing neurons.

Specifically, progenitor cell and radial glial dysfunction emerge as common features of prenatal exposure to drugs of abuse. Alcohol, cocaine and opioids have all been shown to affect the proliferation of progenitor and radial glial cell populations, which are critical for normal cortical development. Additionally, ethanol disrupts radial glial scaffolds essential for neuronal migration.

Neuroinflammation and oxidative stress represent another axis of convergence. All three substances induce a pro-inflammatory environment and increase the production of reactive oxygen species. These processes contribute to an inhospitable developmental environment and likely exacerbate damage initiated by direct neurotoxicity.

Substance-specific mechanisms are also evident to date. Opioids uniquely impact cilial function in the developing brain. Alcohol disrupts radial glial polarity and promotes premature astrocyte formation. Cocaine alters the formation of dopaminergic projections and induces persistent epigenetic changes affecting neuroplasticity systems. While future work will need to thoroughly evaluate how drug-specific such actions are, these distinct findings underscore the value of COs in elucidation of both universal and drug-specific effects of these teratogens.

**Limitations and future directions:** While COs offer numerous advantages over traditional models, they are not without limitations. One of the most prominent is the absence of vasculature. This restricts nutrient and oxygen diffusion in larger organoids and precludes modeling the blood-brain barrier or maternal-fetal exchange. Additionally, organoids generally lack immune cells, such as microglia, unless they are intentionally co-cultured, which limits the study of neuroimmune interactions essential in drug-induced injury.

Temporal limitations are also an important consideration. Most organoids model early to mid-gestational stages, making it challenging to assess their effects on later processes, such as synaptic pruning, myelination, and circuit refinement. Variability in differentiation protocols and organoid architecture further complicates the reproducibility of results across studies.

These limitations notwithstanding, cerebral organoids represent a transformative platform for advancing the study of drugs of abuse that can overcome the limitations of traditional cellular and animal models. The capacity of COs to recapitulate aspects of human neurodevelopment, cellular heterogeneity, and circuit maturation offers unprecedented opportunities to explore how addictive substances shape the developing and mature brain.

Looking forward, COs can serve as powerful models for investigating long-term outcomes of substance exposure, particularly the enduring epigenetic, transcriptomic, and synaptic alterations that may underlie vulnerability to addiction or relapse. Longitudinal CO cultures enable chronic or repeated drug exposure paradigms, permitting the study of adaptive and maladaptive plasticity over extended developmental windows that are not easily accessible in vivo.

Assembloid systems represent a particularly exciting frontier as they integrate multiple region-specific organoids or cell types to reconstitute functional neural circuits. Assembloids connecting cortical, striatal, amygdaloid, and dopaminergic midbrain regions, for instance, provide a physiologically relevant framework for studying addiction-related circuitry such as the mesocorticolimbic pathway. These complex models make it possible to interrogate circuit-level adaptations to addictive substances, including alterations in synaptic connectivity and network oscillations.

Derivation of COs from patient-specific induced pluripotent stem cells facilitates the modeling of individual variability in drug response and susceptibility. Combining organoid models with *in silico* simulations and data from longitudinal birth cohorts could help to link experimental and epidemiological findings. COs will ultimately take their place as a core element of precision medicine in the neuropsychiatric field.

**Conclusion:** Cerebral organoids have already revolutionized our ability to model the effects of prenatal exposure to drugs of abuse in a human-specific, mechanistically rich, and ethically acceptable framework. By revealing both shared and unique pathways of neurotoxicity, organoids offer crucial insights that can inform prevention, policy, and therapy. As these models continue to evolve, they will serve as indispensable tools in the effort to understand and ultimately treat substance-induced neurodevelopmental disorders.

*This work was funded by the Irish Research Council (EPSPG/2022/14) (to LK).*
